# Retrospective analysis of diagnosis and treatment in 9 cases of childhood-onset methylmalonic acidemia in China

**DOI:** 10.3389/fneur.2025.1639775

**Published:** 2025-10-02

**Authors:** Chao Fan, Li Xu, Siyi Gan, Liwen Wu, Haiyan Yang, Zeshu Ning

**Affiliations:** Neurology Department, The Affiliated Children's Hospital of Xiangya School of Medicine, Central South University (Hunan Children’s Hospital), Changsha, Hunan, China

**Keywords:** methylmalonic acidemia, diagnosis, treatment, childhood, gene

## Abstract

**Background:**

Methylmalonic acidemia (MMA) lacks specific clinical manifestations, often leading to misdiagnosis and underdiagnosis by clinicians.

**Methods:**

We retrospectively analyzed the clinical data of children with MMA admitted to the Department of Neurology, Hunan Children’s Hospital, from April 2015 to February 2024.

**Results:**

A total of 9 children with MMA were included. The age at onset ranged from 1 month and 9 days to 8 years, with the time from onset to diagnosis extending up to 1 year and 2 months. Among them, 7 cases were early-onset and 2 were late-onset. Eight cases presented with neurological symptoms, including recurrent seizures, global developmental delay, mental and behavioral abnormalities, and disturbances of consciousness. One case was asymptomatic and confirmed via neonatal screening. Blood biochemistry showed elevated levels of lactic acid, homocysteine, ammonia, alanine aminotransferase, and glucose. All 9 patients received treatments such as vitamin B12 and L-carnitine to improve energy metabolism. Among them, seven achieved favorable clinical outcomes, while two had poor outcomes: one patient with *MMACHA* compound heterozygous variants (c.609G > A/p. Trp203* and c.658-660del/p. Lys220del*) died due to treatment failure, and the other experienced a poor outcome from delayed intervention.

**Conclusion:**

Early-onset MMA is more common, and its clinical manifestations are non-specific. Clinicians should be vigilant about genetic metabolic etiologies in patients with early-onset MMA characterized by recurrent seizures and developmental delay, late-onset MMA with mental and behavioral abnormalities or consciousness disturbances, and those with abnormal metabolic indicators. It is recommended to actively perform blood and urine tandem mass spectrometry and genetic analysis to confirm the diagnosis. The *MMACHA* c.658-660del (p. Lys220del*) variant may be associated with a poor prognosis in MMA patients.

## 1 Introduction

Methylmalonic acidemia (MMA) is the most common autosomal recessive organic acidemia in China, with an overall incidence of approximately 1/50,000–1/100,000 in the general population, and higher incidence in regions with high consanguineous marriage rates (1). It arises from metabolic deficiencies in methylmalonyl-CoA mutase (MCM) or its coenzyme cobalamin (vitamin B₁₂) (1). Based on the age of onset, MMA is classified as early-onset (≤1 year) or late-onset (>1 year). Early-onset MMA is more prevalent, accounting for about 70–80% of childhood-onset cases, characterized by progressive deterioration and high mortality (neonatal-onset cases have an 80% mortality rate), whereas late-onset MMA is rare, with milder clinical manifestations but a risk of misdiagnosis as other neurological disorders (2, 3).

Pathogenetically, MMA is categorized by enzyme defects into MCM deficiency (Mut type, subdivided into Mut0 and Mut- based on enzyme activity) and vitamin B12 metabolic disorder (cbl type, including cblA, cblB, cblC, etc.) (4). Reported MMA-related pathogenic genes include *MMACHC*, *MUT*, *MMAA*, *MMAB*, *MCEE*, *MMADHC*, and others associated with atypical or rare MMA-complicated disorders (e.g., *HCFC1*, *ACSF3*) (4–6). Among these, *MMACHC* mutations are the most common cause of cblC-type MMA in Chinese populations, accounting for over 60% of genetically confirmed cases, while *MUT* mutations are the main cause of Mut-type MMA (5).

In terms of diagnostic technologies, recent advances have significantly improved MMA detection. Newborn screening using tandem mass spectrometry (MS) enables early identification of asymptomatic cases, with studies showing that screened patients have better long-term prognoses due to timely intervention (7, 8). Next-generation sequencing (NGS), including whole-exome sequencing (WES), has become a key tool for genetic confirmation, especially for cases with ambiguous biochemical results, as it can detect pathogenic variants in MMA-related genes with high accuracy (9). However, despite these advances, MMA remains frequently misdiagnosed or underdiagnosed due to its non-specific clinical manifestations—early-onset cases may present with seizures and developmental delay (easily confused with epilepsy or cerebral palsy), while late-onset cases may show mental and behavioral abnormalities (often misdiagnosed as immune-mediated encephalitis) (10, 11).

Against this background, this study summarized and discussed the clinical features, treatment, clinical outcome, and genetic characteristics of 9 children with MMA admitted to our hospital over nearly a decade. The novelty of this study lies in two aspects: first, it focuses on childhood-onset MMA (covering both early and late onset) in a single-center Chinese cohort, providing real-world clinical data that supplements existing epidemiological and clinical studies on MMA in China; second, it identifies a potential prognostic association between the *MMACHC* c.658-660del (p. Lys220del*) variant and poor outcomes, which has not been fully validated in previous small-sample studies. This work aims to help clinicians identify diagnostic clues, make early accurate diagnoses, and ultimately improve patient prognosis—with important implications for reducing medical costs and alleviating social healthcare burdens.

## 2 Methods

### 2.1 Participants

We included children with MMA admitted to the Department of Neurology, Hunan Children’s Hospital, from April 14, 2015, to February 16, 2024. This study was approved by the Ethics Committee of Hunan Children’s Hospital, and informed consent was obtained from the guardians of all participants.

### 2.2 Collection of clinical data

We retrospectively collected clinical data, including general information, clinical symptoms, blood biochemistry results, cranial magnetic resonance imaging (MRI), magnetic resonance angiography (MRA), electroencephalography (EEG), treatment regimens, and clinical outcome.

### 2.3 Blood and urine mass spectrometry

Amino acid and acylcarnitine concentrations in dried blood spots were measured using liquid chromatography–tandem mass spectrometry (LC–MS). Blood sample preparation followed the derivatization method described by Han et al. (12). The tandem mass spectrometers used were API 3200/API 3200MD (SCIEX, Framingham, MA, United States), and the high-performance liquid chromatography system was a SHIMADZU LC-20AD. Urinary organic acid metabolites were quantified via gas chromatography–mass spectrometry (GC–MS) using the method reported by Kimura et al. (13). The mass spectrometer used was a QP-2010 Ultra GC–MS (SHIMADZU, Kyoto, Japan).

### 2.4 WES of peripheral blood

Genomic DNA was extracted from peripheral blood of patients with MMA. WES was performed as described in our previous study (14). Sequence variants were annotated using population and literature databases, including 1,000 Genomes, dbSNP, GnomAD, ClinVar, HGMD, and OMIM. Variant interpretation followed the guidelines of the American College of Medical Genetics and Genomics (ACMG) (9).

## 3 Results

### 3.1 General information

A total of 9 children were included, comprising 4 males and 5 females. The age at onset ranged from 1 month and 9 days to 8 years, with a median age at onset of 5 months and 14 days. The longest time from onset to diagnosis was 1 year and 2 months.

### 3.2 Clinical manifestations

Of the 9 cases, 7 had an onset at < 1 year of age (early-onset). Their main clinical manifestations included recurrent seizures (*n* = 5), global developmental delay (*n* = 1), and asymptomatic presentation (*n* = 1). Blood biochemistry showed elevated homocysteine (*n* = 7), lactic acid (*n* = 7), ammonia (*n* = 5), alanine aminotransferase (*n* = 1), and glucose (*n* = 1). All 7 early-onset cases underwent cranial MRI: 6 showed abnormalities, including subdural effusion (*n* = 4), meningeal thickening with enhancement (*n* = 1), extracerebral space widening (*n* = 4), cerebral atrophy (*n* = 1), bilateral insular patchy long T1/T2 signals (*n* = 1), abnormal white matter signals in bilateral cerebral hemispheres (*n* = 2), and corpus callosum dysplasia (*n* = 1). Complications included a large cerebral venous cisternal cyst (*n* = 1), right lateral ventricle subependymal hemosiderosis (*n* = 1), and 1 case with normal findings. Two children underwent MRA, which showed no abnormalities; MRA results were unavailable for the remaining 5. Among the 7 early-onset cases, 5 underwent EEG (2 had no available EEG results). Of the 5 cases with seizures, 4 underwent EEG, all showing epileptiform discharges; 3 cases had background rhythm slowing, and 1 had no available EEG results.

Two cases had an onset at > 1 year of age (late-onset). One male child (onset at 5 years and 1 month) presented with mental and behavioral abnormalities and disturbances of consciousness. Blood biochemistry showed elevated homocysteine and lactic acid (normal ammonia and glucose). Cranial MRI revealed slightly widened sulci in bilateral cerebral and cerebellar hemispheres, and mild enlargement of bilateral lateral ventricles and the third ventricle. MRA showed sparse branches of bilateral cerebral arteries. EEG showed background rhythm slowing, with widespread spikes and spike-slow waves (predominant in central, parietal, and temporal regions). The other female child (onset at 8 years) presented with dizziness and vomiting. Blood biochemistry showed elevated homocysteine, lactic acid, and ammonia. Cranial MRI revealed slightly widened and deepened sulci in parts of bilateral cerebral hemispheres, with mild ventricular dilatation. MRA showed fine communicating arteries in the right posterior cerebrum. EEG showed multiple slow waves (more prominent on the left).

### 3.3 Mass spectrometry and genetic test results

The blood tandem mass spectrometry of the 9 children all indicated an increase in propionylcarnitine and the ratio of propionylcarnitine to acetylcarnitine. The urine tandem mass spectrometry of all the children also indicated an increase in methylmalonic acid and methylcitric acid, all suggesting methylmalonic acidemia. Four cases underwent genetic testing (Table 1), while results were unavailable for the remaining 5 (Table 2). Genetic mutations identified included two *MUT* variants: c.323G > A (p. R108H) and c.982C > T (p. L328F) (in one early-onset child). Five *MMACHC* variants were identified: c.609G > A (p. Trp203*, *n* = 2), c.658_−_660del (p. Lys220del, *n* = 1), c.217C > T (p. Arg73*, *n* = 1), and c.365A > G (p. His122Arg, *n* = 1); c.217C > T and c.365A > G were found in one late-onset child.

**Table 1:** Clinical characteristics of 4 children with MMA confirmed by gene diagnosis.

Clinical indicators	Case number 2	Case number 4	Case number 6	Case number 8
Sex	M	F	M	M
Age of onset	7 m 12 d	5 m 8 d	5 m 14 d	5 y 1 m
Duration from onset to diagnosis	24 d	1 m	20 d	1 m
Clinical manifestation	Recurrent convulsions	Recurrent convulsions	Recurrent convulsions	Mental and behavioral abnormalities, consciousness disorders
Blood biochemical examination	HCY^#^ 4.10umol/L; LACT^#^ 2.55 mmol/L	HCY^#^ 52.30umol/L; LACT^#^ 3.80 mmol/L; Ammonia^#^ 55.9umol/L	HCY^#^ 30.73umol/L; LACT^#^ 2.15 mmol/L; GLU^#^ 20.98 mmol/L; Ammonia^#^ 85umol/L; ALT^#^ 75.21 IU/L	HCY^#^ 70.1umol/l; LACT^#^ 3.09 mmoL/L
EEG	Background rhythm slows down slightly; Sharp wave and spike wave are distributed in small amount in each region during sleeping period	Slow background rhythm; A small amount of sharp waves, multi spike waves, and sharp spike slow waves are distributed in the occipital and temporal regions	N/A	Slow background rhythm; Widespread spikes and slightly more spikes and slow waves in various regions (central, parietal and temporal regions were significant)
Cranial MRI and MRA	Subdural effusion, meninges thickening and strengthening; MRA: normal	Bilateral insular sac patchy long T1 long T2 signal shadow, bilateral frontotemporal extracerebral space widened with a small subdural effusion; MRA: N/A	Bilateral frontotemporal extracerebral space widened, bilateral frontotemporal subdural effusion; MRA: N/A	Slightly widened sulci in bilateral brain and cerebellar hemispheres, and slightly enlarged bilateral lateral ventricles and third ventricles; MRA: sparse branches of bilateral cerebral arteries
Genetic tests	*MUT* c.323G > A(p. R108H); c.982C > T(p. L328F)	*MMACHC* c.609G > A(p. Trp203*)	*MMACHC* c.609G > A(p. Trp203*); c.658-660del(p. Lys220del*)	*MMACHC* c.217C > T(p. Arg73*); c.365A > G (p. His122Arg)
Treatment	Vitamin B1, vitamin B2, vitamin B6, L-carnitine, levetiracetam	Vitamin B6, vitamin B12, compound vitamin, L-carnitine, levetiracetam	Vitamin B6, vitamin B12, L-carnitine, milk powder with special formula	Vitamin B1, vitamin B2, vitamin B6, vitamin B12, L-carnitine, betaine
Clinical outcome	Seizure free	Seizure free	Death	Normal

ALT, Alanine aminotransferase; d, day; EEG, electroencephalograph; F, Female; GLU, Glucose; HCY, Homocysteine; LACT, Lactic acid; m, month; M, Man; MMA, Methylmalonic acidemia; N/A, Not available.

#, reference range: ALT 0–40 IU/L; HCY 5–15 umol/L; GLU 3.9–6.1 mmol/L; LACT 0.5–2.0 mmol/L; ammonia 20–60 umol/L.

**Table 2:** Clinical characteristics of 5 children with MMA without genetic confirmation.

Clinical indicators	Case number 1	Case number 3	Case number 5	Case number 7	Case number 9
Sex	F	F	F	M	F
Age of onset	2 m 21 d	6 m 15 d	1 m 9 d	5 m	8 y
Duration from onset to diagnosis	10 d	12 d	3 d	1 y 2 m	6 m
Clinical manifestation	Recurrent convulsions	Recurrent convulsions	Asymptomatic	Overall developmental delay	Dizziness, vomiting
Blood biochemical examination	HCY^*^ 33.03umol/L; LACT^*^ 3.36 mmol/L	HCY^*^ 30.81umol/L; LACT^*^ 4.71 mmol/L; ammonia^*^ 114.5umol/L; ALT^*^ 69.20 IU/L	HCY^*^ 23.20umol/L; LACT^*^ 4.11 mmol/L; ammonia^*^ 56.5umol/L	HCY^*^ 115.3umol/L; LACT^*^ 2.64 mmol/L; ammonia^*^ 68.7umol/L	HCY^*^ 82.2umol/L; LACT^*^ 7.32 mmol/L; ammonia^*^ 56.8umol/l
EEG	Normal background rhythm; Occasional sharp waves in the left central and middle temporal regions	Background rhythm slows down slightly; Bilateral occipital and posterior temporal spike waves slightly increased in quantity	N/A	Sharp and slow waves were distributed in the right occipital and posterior temporal regions	Multiple slow waves in each region (significant on the left)
Cranial MRI and MRA	Normal	Bilateral frontotemporal extracerebral space widened, bilateral frontotemporal and anterior longitudinal fissure subdural effusion, brain atrophy; MRA: N/A	Bilateral cerebral hemisphere abnormal white matter signal shadow; MRA: N/A	Abnormal white matter signals in both cerebral hemispheres, corpus callosum dysplasia, slight dilation of ventricular system, lateral fissure and bilateral frontotemporal extracerebral space widened, cerebral venous cisternal cyst, right lateral ventricle subependymal hemosiderosis; MRA: N/A	slight widening and deepening of the sulcus in part of the bilateral cerebral hemispheres, and slight dilatation of the bilateral ventricles; MRA: fine communication arteries in the right cerebral posterior
Genetic tests	N/A	N/A	N/A	N/A	N/A
Treatment	Vitamin B1, vitamin B2, vitamin B6, L-carnitine, levetiracetam	Vitamin B6, vitamin B12, compound vitamin, L-carnitine, levetiracetam	Vitamin B6, vitamin B12, compound vitamin, L-carnitine	Vitamin B6, vitamin B12, compound vitamin, L-carnitine	vitamin B6, vitamin B12, L-carnitine
Clinical outcome	Seizure free	Seizure free	Normal	Developmental delay	Normal

ALT, Alanine aminotransferase; d, day; EEG, electroencephalograph; F, Female; GLU, Glucose; HCY, Homocysteine; LACT, Lactic acid; m, month; M, Man; MMA, Methylmalonic acidemia; N/A, Not available.

*, reference range: ALT 0–40 IU/L; HCY 5–15 umol/L; GLU 3.9–6.1 mmol/L; LACT 0.5–2.0 mmol/L; ammonia 20–60 umol/L.

### 3.4 Treatment and clinical outcome

Seven children with early-onset MMA received vitamins B1, B2, B6, B12, and L-carnitine to improve energy metabolism. Four cases (Cases 1–4) received levetiracetam as an antiepileptic agent, and one case was given a special formula milk powder (without isoleucine, valine, threonine and methionine). Four cases with seizure onset had no recurrence and achieved normal growth and development; 1 asymptomatic case maintained normal growth and development; 1 case remained developmentally delayed; and 1 case died at approximately 1 year and 6 months of age.

Two children with late-onset MMA (Cases 8–9) received vitamins B1, B2, B6, B12, L-carnitine, and betaine to improve energy metabolism, with favorable clinical outcome and full recovery.

## 4 Discussion

Methylmalonic acid is a metabolite of methylmalonyl-CoA in the metabolic pathways of isoleucine, valine, methionine, threonine, cholesterol, and odd-chain fatty acids. Under normal conditions, it is converted to succinate by methylmalonyl-CoA mutase (MCM) and vitamin B12, participating in the tricarboxylic acid cycle. Defects in MCM or vitamin B12 metabolism lead to abnormal accumulation of methylmalonic acid, propionic acid, and methylcitrate, causing multi-organ damage (nervous system, liver, kidneys, bone marrow) (4). Due to non-specific clinical manifestations, MMA diagnosis relies on biochemical (blood/urine MS) and genetic analyses (4).

In this study, we chose WES as the genetic testing method instead of whole-genome sequencing (WGS). Compared with WGS, WES has limitations such as inability to cover large fragment gene deletions/duplications, deep introns, or gene regulatory regions. However, MMA is a known monogenic genetic disorder, and its pathogenic mechanism mainly involves mutations in enzyme-coding genes in the exon regions (9). Additionally, WES has advantages in cost-effectiveness, data interpretation accuracy, and clear recommendations in clinical guidelines (9), which ensures diagnostic efficiency while minimizing over-testing and waste of medical resources.

Clinical severity of MMA varies with age at onset: earlier onset correlates with more severe disease (11). Mut0 type, typically severe and neonatal-onset, presents with acute encephalopathy (vomiting, hypotonia, acidosis, hyperammonemia, coma, seizures) and high mortality (15). Mut-, cblA, cblB, and cblH types are milder, with onset ranging from neonates to adults. Childhood-onset cases are normal at birth, often manifesting within 1 year, with metabolic crises triggered by stress (infection, hunger, vaccines, high-protein diets) - these crises cause intellectual and motor developmental delay and regression, with potential involvement of blood, liver, kidney, skin, and peripheral nerves (15).

In our study, 7 early-onset MMA cases (onset <1 year) presented with recurrent seizures, developmental delay, abnormal metabolic indicators (lactate, glucose, liver enzymes, homocysteine), EEG abnormalities (background slowing, epileptiform discharges), and cranial MRI findings (subdural effusion, meningeal thickening, white matter signals, etc.). These manifestations overlap with common pediatric neurological diseases such as idiopathic epilepsy and hypoxic–ischemic encephalopathy. However, the presence of elevated homocysteine and lactic acid (consistent with metabolic dysfunction) should prompt clinicians to suspect genetic metabolic etiologies, and blood/urine tandem mass spectrometry should be performed promptly. Notably, 1 early-onset case was asymptomatic and detected via neonatal screening—this highlights the value of expanded newborn screening, as it can identify cases before symptom onset and enable early intervention (7, 8).

Late-onset MMA is less common but prone to misdiagnosis. Our two late-onset cases included a 5-year-old with subacute mental and behavioral abnormalities and consciousness disturbances, and an 8-year-old with infection-triggered dizziness and vomiting. The 5-year-old case was initially suspected of having immune-mediated encephalitis (due to subacute onset and mental symptoms), but normal cerebrospinal fluid immune antibodies and elevated homocysteine/lactic acid ruled out this diagnosis. Previous studies have shown that late-onset MMA with mental and behavioral abnormalities should be differentiated from immune-mediated encephalitis: the latter has acute/subacute onset, similar neurological symptoms, but normal metabolic indicators, characteristic MRI signals (cortical and basal ganglia involvement), and positive cerebrospinal fluid/peripheral blood immune antibodies (11). For unclear diagnoses, careful differentiation via blood biochemistry (metabolic indicators) and imaging is essential to avoid excessive testing and reduce medical/family burdens.

MMA treatment includes acute and long-term management (10). Acute treatment involves fluid resuscitation, correcting acidosis/hypoglycemia/electrolyte imbalance, limiting protein intake, and administering L-carnitine and vitamin B12; refractory hyperammonemia and acidosis may require dialysis (10, 16). Long-term treatment differs by vitamin B12 responsiveness: responsive cases (e.g., most cbl types) receive daily 1 mg intramuscular vitamin B12 (hydroxycobalamin preferred), with betaine (100–500 mg/kg/d) for hyperhomocysteinemia, supplemented by L-carnitine (50–100 mg/kg/d), folic acid (5–10 mg/d), and vitamin B6 (10–30 mg/d). Non-responsive cases (e.g., Mut0 type) require a special formula (free of isoleucine, valine, threonine, methionine) with limited natural protein (1.0–2.5 g/kg/d; breast milk preferred for infants), long-term L-carnitine (50–200 mg/kg/d), and monitoring/supplementation of micronutrients due to protein restriction (17, 18). Symptomatic treatment includes antiepileptics (e.g., levetiracetam, as used in our seizure cases) and liver protectants (for organ damage) (19).

Clinical outcome of MMA depends on subtype, onset age, and treatment adherence (1, 3). Vitamin B12-responsive types (especially cblA) have favorable prognoses (70% healthy survival), while non-responsive types (especially Mut0) have poor outcomes (60% mortality, 40% severe developmental delay). Neonatal-onset cases have 80% mortality, while late-onset cases are milder (1, 3). In our study, 7 of 9 cases achieved favorable outcomes, which we attribute to early diagnosis (median time from onset to diagnosis: 24 days) and timely intervention (e.g., vitamin supplementation, antiepileptics, special formula). In contrast, the two poor outcomes were due to either delayed intervention (Case 7, diagnosed 1 year and 2 months after onset, leading to developmental delay) or treatment non-responsiveness (Case 1, died of refractory metabolic complications).

A key finding of our study is the potential association between the *MMACHC* c.658-660del (p. Lys220del*) variant and poor prognosis. Case 6 (who died) carried *MMACHC* compound heterozygous variants (c.609G > A/p. Trp203* and c.658-660del/p. Lys220del*), while Case 4 (with mild developmental delay) carried only the c.609G > A variant. These two cases had similar intervals from onset to diagnosis (20 days vs. 1 months) and comparable initial clinical symptoms (seizures, metabolic acidosis), but drastically different outcomes. Previous literature on the *MMACHC* c.658-660del variant is limited: a 2022 study by Hu et al. reported this variant in 3 of 244 Chinese MMA pedigrees, but did not analyze its prognostic significance (5). Our data suggest that this variant may be a predictor of poor outcome, but further validation in larger cohorts is needed.

Other genetic variants in our study are consistent with previous reports. The *MUT* c.323G > A (p. R108H) and c.982C > T (p. L328F) variants (Case 2) have been classified as LP in ClinVar, and are known to be associated with mild Mut-type MMA (3), which aligns with Case 2’s favorable outcome. The *MMACHC* c.217C > T (p. Arg73*) and c.365A > G (p. His122Arg) variants (Case 8) are common pathogenic variants in Chinese cblC-type MMA patients (5), and Case 8’s favorable clinical outcome is consistent with the generally favorable prognosis of late-onset cblC-type MMA (20).

## 5 Conclusion

In summary, MMA lacks specific clinical manifestations, with early-onset cases predominating and high misdiagnosis risk. Clinicians should suspect genetic metabolic causes in children <1 year with seizures and developmental delay, abnormal metabolic indicators (homocysteine, lactic acid, ammonia), or late-onset cases with mental and behavioral abnormalities. Prompt blood and urine mass spectrometry (for biochemical confirmation) and genetic testing (for subtype classification) are recommended to guide treatment. Advances in diagnostic technologies (e.g., newborn screening, WES) have enabled earlier intervention, which is critical for improving long-term outcomes. The *MMACHC* c.658-660del (p. Lys220del*) variant may be associated with a poor prognosis in MMA patients, but further studies are needed to validate this finding.

## 6 Limitations

This study has several limitations that should be acknowledged: (1) Small sample size: Only 9 cases were included, which may limit the generalizability of our findings (especially the prognostic association of the MMACHC c.658-660del variant). Future multi-center studies with larger cohorts are needed to validate these results. (2) Single-center and retrospective design: The study was conducted at a single children’s hospital, and the retrospective nature may have led to missing data (e.g., incomplete genetic testing in 5 cases, lack of long-term follow-up in some cases). Prospective studies would provide more reliable data on clinical outcomes. (3) Incomplete genetic testing: Genetic results were unavailable for 5 cases, which prevented a comprehensive analysis of genotype–phenotype correlations. Additionally, WES may have missed large fragment deletions/duplications or intronic variants, which could be detected by WGS or copy number variation (CNV) analysis. (4) Limited follow-up duration: The median follow-up time was 3 months, and long-term outcomes (e.g., neurocognitive development in late childhood, renal function in adulthood) were not evaluated. Longer follow-up is needed to assess the long-term impact of MMA and its treatment.

## Data Availability

The raw data supporting the conclusions of this article will be made available by the authors, without undue reservation.
